# Receptor tyrosine kinase expression of circulating tumor cells in small cell lung cancer

**DOI:** 10.18632/oncoscience.179

**Published:** 2015-07-31

**Authors:** Gerhard Hamilton, Barbara Rath, Lukas Klameth, Maximilian Hochmair

**Affiliations:** ^1^ Ludwig Boltzmann Cluster of Translational Oncology, Vienna, Austria; ^2^ Respiratory Oncology Unit, Otto Wagner Spital, Vienna, Austria

**Keywords:** Small cell lung cancer, circulating tumor cells, receptor tyrosine kinases, cancer stem cells, Wnt Pathway

## Abstract

Small cell lung cancer (SCLC) has a poor prognosis and is found disseminated at first presentation in the majority of cases. The cell biological mechanisms underlying metastasis and drug resistance are not clear. SCLC is characterized by high numbers of circulating tumor cells (CTCs) and we were able to expand several CTC lines *ex vivo* and to relate chitinase-3-like-1/YKL-40 (CHI3L1) as marker. Availability of expanded SCLC CTC cells allowed for a screening of receptor tyrosine kinases (RTKs) expressed. The metastatic CHI3L1-negative SCLC cell line SCLC26A, established from a pleural effusion was used for comparison. The CTC cell line BHGc10 was found to exhibit increased expression of RYK, AXL, Tie-1, Dtk, ROR1/2, several ephrins (Eph) and FGF/EGF receptors compared to SCLC26A. All of these RTKs have been associated with cell motility, invasion and poor prognosis in diverse cancer entities without knowledge of their association with CTCs. The identification of RYK, AXL and ROR1/2 as pseudokinases, lacking activity, seems to be related to the observed failure of RTK inhibitors in SCLC. These kinases are involved in the noncanonical WNT pathway and their expression in SCLC CTCs represents a cancer stem cell-like phenotype.

## INTRODUCTION

Lung cancer is the leading cause of tumor-associated death worldwide [1]. Small cell lung cancer (SCLC) is a neuroendocrine and highly malignant subtype of lung cancer, accounting for approximately 15% of cases, which exhibits a low survival rate at disseminated stage. In contrast to non-small lung cancer (NSCLC), targeted therapy directed to specific oncogenes is not available for SCLC and platinum-based combination chemotherapy with etoposide and second-line topotecan are standard care [2]. SCLCs invariably show inactivation of p53 and retinoblastoma, and in the absence of these two tumor suppressors several alternative growth factor pathways induce vigorous progression [3]. Despite an initial high response rate, tumors relapse early and are not amenable to effective further therapy. Progress of research on SCLC is hampered by availability of tumor material, as in extended disease no material is collected after initial confirmation of the diagnosis by fine-needle biopsies [4]. Alternatively, so-called liquid biopsies consist of the detection of circulating genetic material or enrichment of the diversified circulating tumor cells (CTCs) using blood samples [5]. In most cases CTCs are scarce and investigations are restricted to single-cell genetic analysis, detection of surface markers or characterization of a limited number of cytokines by the EPISPOT assay which detects proteins secreted/released/shed from single epithelial cancer cells [6]. SCLC is set apart from other cancers by extremely high number of CTCs in part of the patients [7]. This allowed us for the first time to expand SCLC *ex vivo* and to set up a few CTC cell lines [8]. So far, several CTC lines have been obtained in breast cancer and one in colon cancer, respectively [9, 10]. Screening of the CTC cell culture supernantants for cytokines/chemokines revealed expression of chitinase-3-like-1 (CHI3L1)/YKL-40 as CTC marker [11]. In the present work we examined the BHGc10 CTC line for the presence of one or several receptor tyrosine kinases (RTKs) in consideration of the detection of putative new therapeutic targets. To our knowledge the present work is the first report on expression of RTKs in a pure population of CTCs.

## RESULTS

**Figure 1:**
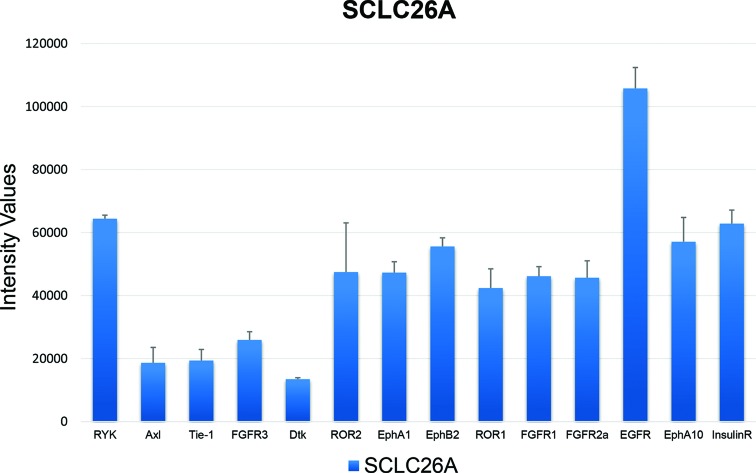
Expression of selected RTKs in SCLC26A cells RTKs expressed in SCLC26A cAQ1ells which correspond to RTKs overexpressed in BHGc10 CTC cells are shown (mean ± SD, arbitrary intensity units).

Expression of RTKs was detected on the Western blot arrays for SCLC26A and BHGc10 SCLC CTC cells, respectively. Figure 1 shows the control RTKs of the local metastatic SCLC26A cell line to be compared with those of the BHGc10 SCLC CTC line, isolated from peripheral blood. SCLC26A relies on EGFR, InsulinR and FGFRs for growth and expresses several kinases, including RYK, ROR1/2 and members of the ephrin family.

**Figure 2:**
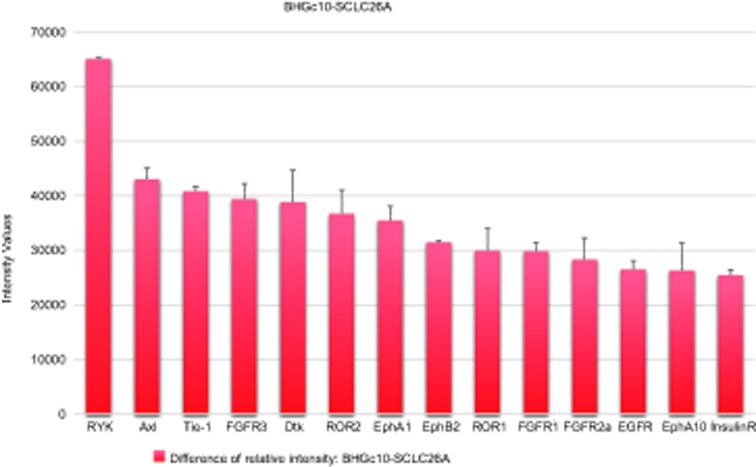
Overexpression of selected RTKs in BHGc10 CTC cells The figure shows the increase of expression of the most altered corresponding RTKs in the CTC cell line (mean ± SD, identical arbitrary intensity units).

Figure 2 lists the RTKs which exhibited the largest increase in this CHI3L1-positive CTC cell line, sorted according to the differences in relation to SCLC26A. Relative overexpression in the CTC cells was detected for RYK, AXL, Tie-1, Dtk, ROR1/2, and Ephrins EphA1 and EphB2. Growth factor receptors FGFR1 and FGFR2a were increased, as well as EGFR and InsulinR. Minor decreases were seen for PDGFRa, Flt-3, ephrins EphA6 and EphA7, MSPR and TrkA (data not shown).

## DISCUSSION

In general, CTCs are relatively rare cells but access to *in vitro* expanded CTCs renders analysis of the cell biologic characteristics possible [8]. Additionally, the expanded BHGc10 SCLC CTC cells lack distinct expression of some SCLC markers and would have possibly not selected by commonly applied enrichment methods relying on cell surface markers (data not shown). Human Proteome Profiler arrays were used to compare the relative expression of RTKs of SCLC26A and BHGc10 SCLC CTCs, representing progression from local metastasis to the disseminated state in peripheral blood.

The RTK family regulates a wide spectrum of cell activities including proliferation, differentiation, metabolism and migration [14]. Aberrant RTK activation due to deregulated receptor expression and/or constitutive activation are major mechanisms by which tumor cells proliferate and spread. RTKs are type I transmembrane proteins with an extracellular ligand-binding region, a single-pass transmembrane coil and an intracellular protein tyrosine kinase domain adjacent to regulatory sequences [14]. Interestingly, approximately 10% of the more than 500 human kinases are pseudokinases which lack catalytic residues within their kinase domain [15]. ErbB3 constitutes a prototype of catalytically inactive RTK function, adopting the role of signal modulation in heterodimeric receptor complexes with other ErbB subfamily members [14].

RYK (for related to tyrosine kinase) has been isolated from a DNA library of SKOV-3, an epithelial ovarian cancer cell line [16]. Localization of RYK mRNA in the epithelial and stromal compartment of tissues such as brain, lung, colon, kidney, and breast was observed [17]. RYK is an unusual member of the RTK family classified as a putative pseudokinase. RYK regulates basic biological processes in response to the high affinity binding of Wnt family ligands to its extracellular Wnt inhibitory factor (WIF) domain [18, 19]. Upon Wnt binding, RYK participates in the activation of β-catenin–dependent or –independent signaling pathways controling cell polarity, cell differentiation and cell migration. Additonally, RYK can signal via activation of the small GTPase RhoA [20]. Many RYK-interactive partners, such as Mindbomb 1, an E3 ubiquitin ligase, cell polarity protein Vangl2 and ephrin receptor have been identified but they do not represent suitable targets for antagonizing RYK [21 - 23].

RYK overexpression has been observed in human ovarian cancer and was correlated with decreased overall survival of patients [16, 24]. Overexpression of RYK is transforming *in vitro* and *in vivo* [25]. The RYK receptor has been localized in malignant epithelium and blood vessel, but no correlation was found with age, histological subtype, degree of differentiation, FIGO stage, or residual disease in ovarian cancer patients. Immunoreactivity was also present in the stromal tissue of most tumors, predominantly in the desmoplastic tissue of carcinomas, partially assigned to lymphocytic inflammatory cells. Pediatric brain tumors derived from neuroepithelial precursor cells express RYK [26]. Targeted inhibition of RYK function with conventional small molecule ATP-competitive RTK inhibitors seems not feasible.

ROR1/2 are a single-span transmembrane RTKs which function in mediating the non-canonical Wnt signaling and has no essential roles known in adult tissues [27]. ROR2 was first identified as a RTK-like orphan receptor and demonstrated to be critical for filopodia formation during cell migration, a process dependent on Wnt5a. Wnt signaling plays important roles in lung development and lung cancer. ROR2 was found to be highly expressed in osteosarcoma, renal cell carcinomas, melanoma, colon cancer, squamous cell carcinoma of the head and neck, and breast cancer [28]. ROR2 is expressed in the majority of breast cancer patients, but not in normal breast tissue, and this expression confers a poorer disease-specific survival for these patients [29]. Generally, ROR2 expression is associated with more aggressive disease states. Wnt5a and Wnt3a are now well known to act as ligands for ROR2 to activate Wnt signaling activity. Thus, ROR2 mediates polarized cell migration, invasion, and tumor growth; however, the mechanism by which ROR2 functions to promote cancer remained elusive [30]. The association of ROR2 with Twist and MMP2 indicated a role as a regulator of EMT and cell migration [31].

ROR2 has clearly been linked to aggressive disease patterns and has been implicated in important tumor promoting signaling pathways constituting an promising target. As a protein ROR2 is not normally expressed in adult tissues. There are several drugs targeting activated RTKs for treatment of cancers, with particular efficacy for the small-molecule inhibitors that target the ATP-binding site of the intracellular tyrosine kinase domain but these will not work for pseudokinases [32]. ROR2 is a cell surface receptor with a distinct extracellular domain and a suitable target for monoclonal antibodies for therapeutic use and such antibodies can function to neutralize the receptor or can mediate antibody-dependent cytotoxicity [33].

The TYRO3, AXL and MERTK (TAM) family of RTKs are aberrantly expressed in multiple hematological and epithelial malignancies. Their induction in tumor cells predominately promotes survival, chemoresistance and motility [34]. Although MERTK and AXL can activate standard proliferative pathways (ERK, AKT and members of the STAT family), their presence generally promotes survival. AXL functions together with the type I interferon receptor to increase suppressor of cytokine signaling 1 (SOCS1) and SOCS3 expression, which helps to turn down inflammatory toll-like receptor signaling. Furthermore, MERTK and AXL decrease NK cell antitumor activity. Hypoxic conditions in the tumor increase the expression of HIF1α, which directly promotes transcription of AXL. Inhibition of AXL reversed chemoresistance by decreasing expression of the anti-apoptotic proteins BCL-2, BCL-XL, MCL1 and survivin in tumor cells and activated pro-apoptotic proteins BAD, BAX, BCL-2, BAK and PUMA. AXL in particular has been implicated in metastasis in multiple tumor types and facilitates EMT in addition to upregulation of MMP9 [34]. The angiopoietin-Tie signaling system is a vascular-specific RTK pathway that is essential for normal vascular development and is elevated in many cancers and inflammation [35]. Signaling by Tie receptors appears to complement the VEGF pathway. Developmental tyrosine kinase (Dtk) encodes a protein with structural similarities to AXL and is expressed in a number of leukemic cell lines [36].

Eph receptors are the largest family of RTKs [37]. Reemergence of their embryonic functions in cancers is implicated in tumor progression, neoangiogenesis and metastasis of a large range of epithelial and mesenchymal malignancies. Eph receptors mediate cell-cell interaction both in tumor cells and in tumor microenvironment, namely the tumor stroma and tumor vasculature [38, 39]. Ephrins are expressed at the leading edge of glioblastoma cells invasion into the brain parenchyma. Eph targeting studies in preclinical models of GBM have been very encouraging and may provide the means to treat these highly refractory aggressive tumors [40]. A host of therapeutic approaches including small-molecule kinase inhibitors, ephrin-mimetic peptides, peptide vaccines, short hairpin RNAs, function-modulating fusion proteins and monoclonal antibodies have been developed and are at different stages of preclinical and clinical evaluation.

Cancer stem cells (CSCs) have been increasingly described for many malignancies. CSCs display many features of embryonic or tissue stem cells, and feature persistent activation of one or more conserved signal transduction pathways involved in development and tissue homeostasis, such as the Notch, Hedgehog and Wnt pathways [41]. Wnt signals are transduced to the noncanonical pathway through frizzled family receptors and ROR2/RYK coreceptors to the dishevelled-dependent or the Ca2+-dependent signaling cascades for control of cell movement and tissue polarity [42]. In lung cancer, Wnt signaling substantially impacts NSCLC tumorigenesis, prognosis, and resistance to therapy [43, 44]. The Wnt5a ligand can induce hematopoietic stem cell (HSC) quiescence through a noncanonical Wnt pathway, resulting in an increased ability to reconstitute hematopoiesis. Inhibition of RYK blocked the ability of Wnt5a to induce HSC quiescence and to enhance short-term and long-term hematopoietic repopulation [45]. Wnt5a RYK-dependently suppressed production of reactive oxygen species, a known inducer of HSC proliferation.

Generation of CSCs is linked to EMT and are capable of tumor initiation and self-renewal, contributing to treatment resistance and metastases [46]. AXL is acting to induce EMT in epithelial cells to regulate self-renewal and chemoresistance and its expression is associated with the expression of stem cell and metastases genes and increased tumorigenicity through promotion of invasion and migration. Downregulation of AXL using MP470 (Amuvatinib) reversed EMT in mesenchymal normal human mammary epithelial cells and in a clinical study, Amuvatinib was well tolerated and showed antitumor activity when combined with paclitaxel/carboplatin and carboplatin/etoposide in neuroendocrine, NSCLC, and SCLC tumors [47].

In conclusion, RTK expression of a SCLC CTC line was here delineated for the first time. The overexpression of specific RTKs in a blood-derived line versus a metastase-derived SCLC line is in line with use of Wnt signaling, identifying this CTC line as CSC-related. The RTK pseudokinases found seem to escape targeting by RTK inhibitors but may be tackled by other means. Especially AXL may be related to the high degree of chemoresistance, observed in relapsing SCLC patients. Since the respective kinases expressed in the SCLC CTC cell line were broadly found in other tumors, this model of dissemination may hold true for other malignancies as well.

## MATERIALS AND METHODS

### Cell lines

SCLC26A cell line was established in our lab as well as the BHGc10 SCLC CTC line, derived from a peripheral blood sample of a relapsed SCLC patient [8, 12]. For the detection of RTKs, cells were cultured in RPMI-1640 medium (Sigma-Aldrich, St. Louis, MO, USA), supplemented with 10% bovine serum (Seromed, Berlin, Germany) and antibiotics (Sigma-Aldrich; Penicillin/Streptomycin/Neomycin solution) under tissue culture conditions.

### Screening of cells for expression of RTKs

RTKs were detected using a Western Blot array (Proteome Profiler Human Phospho-RTK Array Kit, ARY001B, R&D Systems, Minneapolis, MN, USA) according to the manufacturer's instructions. The experiments were done in duplicate. In brief, 1×107 cells/ml were washed in PBS and extracted using the appropriate buffer of the kit supplemented with protease inhibitor cocktail (Sigma-Aldrich). Extracts were centrifuged, supernantants diluted with sample buffer and applied to the nitrocellulose membranes having 49 anti-kinase catcher antibodies spotted. RTKs were finally detected using pan-anti-tyrosine-HRP conjugate and detection of binding by chemiluminescence. Spots were analyzed using GelAnalyzer [13] and ORIGIN 9.0 software (OriginLab, Northampton, MA, USA).

## References

[1] Siegel RL, Miller KD, Jemal A (2015). Cancer statistics, 2015. CA Cancer J Clin.

[2] Hensing T, Chawla A, Batra R, Salgia R (2014). A personalized treatment for lung cancer: molecular pathways, targeted therapies, and genomic characterization. Adv Exp Med Biol.

[3] Pietanza MC, Ladanyi M (2012). Bringing the genomic landscape of small-cell lung cancer into focus. Nat Genet.

[4] Stamatis G (2014). Neuroendocrine tumors of the lung: the role of surgery in small cell lung cancer. Thorac Surg Clin.

[5] Rolfo C, Castiglia M, Hong D, Alessandro R, Mertens I, Baggerman G, Zwaenepoel K, Gil-Bazo I, Passiglia F, Carreca AP, Taverna S, Vento R, Peeters M (2014). Liquid biopsies in lung cancer: the new ambrosia of researchers. Biochim Biophys Acta.

[6] Alix-Panabières C (2012). EPISPOT assay: detection of viable DTCs/CTCs in solid tumor patients. Recent Results Cancer Res.

[7] Yu N, Zhou J, Cui F, Tang X (2015). Circulating tumor cells in lung cancer: detection methods and clinical applications. Lung.

[8] Hamilton G, Burghuber O, Zeillinger R Circulating tumor cells in small cell lung cancer: ex vivo expansion. Lung.

[9] Yu M, Bardia A, Aceto N, Bersani F, Madden MW, Donaldson MC, Desai R, Zhu H, Comaills V, Zheng Z, Wittner BS, Stojanov P, Brachtel E (2014). Ex vivo culture of circulating breast tumor cells for individualized testing of drug susceptibility. Science.

[10] Cayrefourcq L, Mazard T, Joosse S, Solassol J, Ramos J, Assenat E, Schumacher U, Costes V, Maudelonde T, Pantel K, Alix-Panabières C (2015). Establishment and characterization of a cell line from human circulating colon cancer cells. Cancer Res.

[11] Hamilton G, Rath B, Burghuber O (2015). Chitinase-3-like-1/YKL-40 as marker of circulating cancer cells. Transl Lung Cancer Res.

[12] Hamilton G, Klameth L, Rath B, Thalhammer T (2014). Synergism of cyclin-dependent kinase inhibitors with camptothecin derivatives in small cell lung cancer cell lines. Molecules.

[13] Lazer I, Lazer I (2010). GelAnalyzer 2010a: Freeware 1D gel electrophoresis image analysis software. http://www.gelanalyzer.com.

[14] Lemmon MA, Schlessinger J (2010). Cell signaling by receptor tyrosine kinases. Cell.

[15] Zhang H, Photiou A, Grothey A, Stebbing J, Giamas G (2012). The role of pseudokinases in cancer. Cell Signal.

[16] Wang XC, Katso R, Butler R, Hanby AM, Poulsom R, Jones T, Sheer D, Ganesan TS (1996). H-RYK, an unusual receptor kinase: isolation and analysis of expression in ovarian cancer. Mol Med.

[17] Hovens CM, Stacker SA, Andres AC, Harpur AG, Ziemiecki A, Wilks AF (1992). RYK, a receptor tyrosine kinase-related molecule with unusual kinase domain motifs. Proc Natl Acad Sci. USA.

[18] Halford MM, Stacker SA (2001). Revelations of the RYK receptor. Bioessays.

[19] Hsieh JC, Kodjabachian L, Rebbert ML, Rattner A, Smallwood PM, Samos CH, Nusse R, Dawid IB, Nathans J (1999). A new secreted protein that binds to Wnt proteins and inhibits their activities. Nature.

[20] Macheda ML, Sun WW, Kugathasan K, Hogan BM, Bower NI, Halford MM, Zhang YF, Jacques BE, Lieschke GJ, Dabdoub A, Stacker SA (2012). The Wnt receptor Ryk plays a role in mammalian planar cell polarity signaling. J Biol Chem.

[21] Berndt JD, Aoyagi A, Yang P, Anastas JN, Tang L, Moon RT (2011). Mindbomb 1, an E3 ubiquitin ligase, forms a complex with RYK to activate Wnt/β-catenin signaling. J Cell Biol.

[22] Andre P, Wang Q, Wang N, Gao B, Schilit A, Halford MM, Stacker SA, Zhang X, Yang Y (2012). The Wnt coreceptor Ryk regulates Wnt/planar cell polarity by modulating the degradation of the core planar cell polarity component Vangl2. J Biol Chem.

[23] Halford MM, Armes J, Buchert M, Meskenaite V, Grail D, Hibbs ML, Wilks AF, Farlie PG, Newgreen DF, Hovens CM, Stacker SA (2000). Ryk-deficient mice exhibit craniofacial defects associated with perturbed Eph receptor crosstalk. Nat Genet.

[24] Katso RM, Manek S, Ganjavi H, Biddolph S, Charnock MF, Bradburn M, Wells M, Ganesan TS (2000). Overexpression of H-Ryk in epithelial ovarian cancer: prognostic significance of receptor expression. Clin Cancer Res.

[25] Katso RM, Manek S, Biddolph S, Whittaker R, Charnock MF, Wells M, Ganesan TS (1999). Overexpression of H-Ryk in mouse fibroblasts confers transforming ability in vitro and in vivo: correlation with up-regulation in epithelial ovarian cancer. Cancer Res.

[26] Weiner HL, Rothman M, Miller DC, Ziff EB (1996). Pediatric brain tumors express multiple receptor tyrosine kinases including novel cell adhesion kinases. Pediatr Neurosurg.

[27] Li C, Chen H, Hu L, Xing Y, Sasaki T, Villosis MF, Li J, Nishita M, Minami Y, Minoo P (2008). Ror2 modulates the canonical Wnt signaling in lung epithelial cells through cooperation with Fzd2. BMC Mol Biol.

[28] Debebe Z, Rathmell WK (2015). Ror2 as a Therapeutic Target in Cancer. Pharmacol Ther.

[29] Henry C, Quadir A, Hawkins NJ, Jary E, Llamosas E, Kumar D, Daniels B, Ward RL, Ford CE (2015). Expression of the novel Wnt receptor ROR2 is increased in breast cancer and may regulate both β-catenin dependent and independent Wnt signalling. J Cancer Res Clin Oncol.

[30] Morioka K1, Tanikawa C, Ochi K, Daigo Y, Katagiri T, Kawano H, Kawaguchi H, Myoui A, Yoshikawa H, Naka N, Araki N, Kudawara I, Ieguchi M (2009). Orphan receptor tyrosine kinase ROR2 as a potential therapeutic target for osteosarcoma. Cancer Sci.

[31] Wright TM, Brannon AR, Gordan JD, Mikels AJ, Mitchell C, Chen S, Espinosa I, van de Rijn M, Pruthi R, Wallen E, Edwards L, Nusse R, Rathmell WK (2009). Ror2, a developmentally regulated kinase, promotes tumor growth potential in renal cell carcinoma. Oncogene.

[32] Maina F (2014). Strategies to overcome drug resistance of receptor tyrosine kinase addicted cancer cells. Curr Med Chem.

[33] Halford MM, Macheda ML, Parish CL, Takano EA, Fox S, Layton D, Nice E, Stacker SA (2013). A fully human inhibitory monoclonal antibody to the Wnt receptor RYK. PLoS One.

[34] Graham DK, DeRyckere D, Davies KD, Earp HS (2014). The TAM family: phosphatidylserine sensing receptor tyrosine kinases gone awry in cancer. Nat Rev Cancer.

[35] Thurston G, Daly C (2012). The complex role of angiopoietin-2 in the angiopoietin-tie signaling pathway. Cold Spring Harb Perspect Med.

[36] Crosier PS, Hall LR, Vitas MR, Lewis PM, Crosier KE (1995). Identification of a novel receptor tyrosine kinase expressed in acute myeloid leukemic blasts. Leuk Lymphoma.

[37] Boyd AW, Bartlett PF, Lackmann M (2014). Therapeutic targeting of EPH receptors and their ligands. Nat Rev Drug Discov.

[38] Lim JJ, Xie D (2013). The roles and therapeutic potentials of Ephs and ephrins in lung cancer. Exp Cell Res.

[39] Brantley-Sieders D, Schmidt S, Parker M, Chen J (2004). Eph receptor tyrosine kinases in tumor and tumor microenvironment. Curr Pharm Des.

[40] Day BW, Stringer BW, Boyd AW (2014). Eph receptors as therapeutic targets in glioblastoma. Br J Cancer.

[41] Takebe N, Miele L, Harris PJ, Jeong W, Bando H, Kahn M, Yang SX, Ivy SP (2015). Targeting Notch, Hedgehog, and Wnt pathways in cancer stem cells: clinical update. Nat Rev Clin Oncol.

[42] Katoh M, Katoh M (2007). WNT signaling pathway and stem cell signaling network. Clin Cancer Res.

[43] Stewart DJ (2014). Wnt signaling pathway in non-small cell lung cancer. J Natl. Cancer Inst.

[44] Bafico A, Liu G, Goldin L, Harris V, Aaronson SA (2004). An autocrine mechanism for constitutive Wnt pathway activation in human cancer cells. Cancer Cell.

[45] Povinelli BJ, Nemeth MJ (2014). Wnt5a regulates hematopoietic stem cell proliferation and repopulation through the Ryk receptor. Stem Cells.

[46] Asiedu MK, Beauchamp-Perez FD, Ingle JN, Behrens MD, Radisky DC, Knutson KL (2014). AXL induces epithelial-to-mesenchymal transition and regulates the function of breast cancer stem cells. Oncogene.

[47] Mita M, Gordon M, Rosen L, Kapoor N, Choy G, Redkar S, Taverna P, Oganesian A, Sahai A, Azab M, Bristow R, Tolcher AW (2014). Phase 1B study of amuvatinib in combination with five standard cancer therapies in adults with advanced solid tumors. Cancer Chemother Pharmacol.

